# Bone marrow mesenchymal stem cell-derived exosomes promote osteoblast proliferation, migration and inhibit apoptosis by regulating KLF3-AS1/miR-338-3p

**DOI:** 10.1186/s12891-024-07236-0

**Published:** 2024-02-09

**Authors:** Dacheng Liu, Xuechao Zhao, Qiang Zhang, Fei Zhou, Xiangyang Tong

**Affiliations:** 1 Department of Orthopedics, Xuzhou Municipal Hospital Affiliated to Xuzhou Medical University, 269 University Road, Tongshan District, Xuzhou, 221100 Jiangsu China; 2 Operating Room, Xuzhou Central Hospital, Xuzhou, 221006 China

**Keywords:** Bone marrow mesenchymal stem cells, Exosomes, KLF3-AS1, miR-338-3p

## Abstract

**Aim:**

This study aimed to investigate the effect and mechanism of bone marrow mesenchymal stem cell-derived exosomes on osteoblast function.

**Methods:**

The expression of KLF3-AS1 and miR-338-3p in serum of fracture patients was detected by qRT-PCR. Exosomes from BMSCs were isolated by ultrafast centrifugation. MC3T3-E1 cells were cultured in vitro as experimental cells. Intracellular gene expression was regulated by transfection of si-KLF3-AS1 or miR-338-3p inhibitors. MTT assay, Transwell assay and flow cytometry were used to evaluate cell viability, migration, and apoptosis. The luciferase reporter gene was used to verify the targeting relationship between KLF3-AS1 and miR-338-3p. Bioinformatics analysis was used to identify the basic functions and possible enrichment pathways of miR-338-3p target genes.

**Results:**

The expressions of KLF3-AS1 and miR-338-3p in the serum of fracture patients were down-regulated and up-regulated, respectively. The expression of KLF3-AS1 was increased in MC3T3-E1 cells cultured with BMSCs-Exo, while the viability and migration ability of MC3T3-E1 cells were enhanced, and the apoptosis ability was weakened. Further analysis revealed miR-338-3p was the target gene of KLF3-AS1. The expression of miR-338-3p was downregulated in MC3T3-E1 cells cultured with BMSCs-Exo. Inhibition of miR-338-3p in MC3T3-E1 cells enhanced the viability and migration ability of MC3T3-E1 cells when cultured with BMSCs-Exo, while suppressing apoptosis. Bioinformatics analysis demonstrated that the target genes of miR-338-3p were predominantly localized at the axon’s initiation site, involved in biological processes such as development and growth regulation, and mainly enriched in MAPK and ErbB signaling pathways.

**Conclusion:**

In vitro, BMSCs-Exo exhibits the capacity to enhance proliferation and migration while inhibiting apoptosis of MC3T3-E1 cells, potentially achieved through modulation of KLF3-AS1 and miR-338-3p expression in MC3T3-E1 cells.

## Introduction

Fracture repair is a regeneration process, including angiogenesis, stem cell differentiation, osteogenesis, and cartilage formation [1]. Large-scale bone defect caused by fracture is easy to induce delayed union or even nonunion of fracture, which brings great pain and inconvenience to clinical patients [2]. In an aging society, the ability of fracture healing is gradually declining, and the extension of rehabilitation process after fracture may lead to serious complications. Therefore, it is very important to promote bone regeneration, bone formation and calcification during the fracture healing process.

Exosomes are vesicle structures secreted by cells, characterized by the encapsulation of various small molecules such as mRNA, miRNA and proteins within lipid bilayers [3]. Exosomes serve as intercellular communication mediators and play a crucial role in repairing lesions similar to or even surpassing that of mesenchymal stem cells (MSCs) through various signal transduction pathways [4]. Bone marrow MSCs (BMSCs), capable of differentiating into osteoblasts, epidermal cells and adipocytes, are widely used in cell therapy and tissue repair [5]. Previous studies have shown that BMSCs transplantation promotes fracture healing and exhibits significant therapeutic effects on myocardial injury, articular cartilage injury and osteoarthritis [6]. Moreover, BMSCs release chemokines, cytokines or growth factors to stimulate target cells for paracrine-mediated release of functionally active mediators which contribute to endogenous regeneration. The objective of this study was to elucidate the role of exosomes-derived BMSCs in fracture healing.

Long non-coding RNA (lncRNA) is a kind of RNA molecules with little or no protein coding ability, and a transcript length is over 200nt. It is located in the nucleus or cytoplasm and regulates gene expression at the level of epigenetics, transcriptional regulation and post-transcriptional regulation [7]. More and more evidence showed that lncRNA is involved in osteogenesis and cartilage differentiation. For example, it was found that H19 was involved in osteogenic differentiation of stem cells by regulating the Wnt signal pathway [8]. Recent studies have shown that BMSC-derived exosomes (BMSCs-Exo) contain a large amount of lncRNA KLF3-AS1 (Krüppel-like factor 3 antisense RNA 1), which can promote the proliferation of chondrocyte in vitro [9]. At present, the function of KLF3-AS1 in osteoblasts is still unclear. MicroRNAs (miRNAs) are a class of single-stranded non-coding RNAs with a length of 19-25nt [10]. LncRNA is usually a competing endogenous RNA (ceRNA) of miRNA, which reduces the inhibitory function of miRNA on target genes and thus plays a role in gene regulation. Bioinformatics analysis showed that KLF3-AS1 was a ceRNA of miR-338-3p [11]. MiR-338-3p is located on chromosome 17q25.3 with a length of 22nt. Tong et al. found that the dysregulation of miR-338-3p was related to age-related osteoporosis [12]. Liu et al. reported that the expression of miR-338-3p decreased during osteogenic differentiation of BMSCs [13]. Therefore, we speculated that both KLF3-AS1 and miR-338-3p could play certain roles in osteoblasts.

On the basis of the above studies, this study obtained BMSCs-derived exosomes (BMSCs-Exo) and attempted to explore the effects of BMSCs-Exo on proliferation, migration and apoptosis of osteoblasts and its possible molecular mechanism through in vitro experiments.

## Materials and methods

### Study population and serum samples

This study was approved by the Ethics Committee of Xuzhou Municipal Hospital Affiliated to Xuzhou Medical University, and all subjects gave informed consent. A total of 65 patients with fractures were included in this study, including 39 males and 26 females, with an average age of (48.11 ± 4.34) years. According to the location of the fracture site, the patients were divided into intra-articular fracture (*n* = 30) and hand fracture (*n* = 35). During the same time, 65 healthy volunteers were recruited in the physical examination center as the control group, including 38 males and 27 females, with an average age of (47.71 ± 4.27) years. No autoimmune disease or inflammatory disease was found in both groups. All subjects were required to collect fasting venous blood on the next day after enrollment and isolate serum for reserve use.

### Cell culture and treatment

Mouse osteogenic cell line MC3T3-E1, purchased from American Type Culture Collection (ATCC), was cultured with DMEM medium containing 10% fetal bovine serum (FBS) and 1% penicillin/streptomycin. MC3T3-E1 cells were suitable for growth in a humidity adjustable environment containing 5% CO_2_ at 37°C. Primary mouse bone marrow mesenchymal stem cells (BMSCs) were purchased from ATCC and cultured in a humidity-adjustable incubator containing 5% CO_2_ at 37°C.

### Extraction and identification of exosomes derived from BMSCs

Exosomes derived from BMSCs (BMSCs-Exo) were extracted based on previously published methods [14]. P3-P5 generation BMSCs with good growth state and fusion to about 80% were selected, washed twice with PBS, and replaced with complete culture medium containing serum without exosome. After 24 h of cell culture, the cell supernatants were collected and centrifuged at 300 g for 10 min, 2000 g for 10 min and 10,000 g for 30 min at 4°C to remove impurities such as cell debris and organelles. The supernatant was transferred to the ultra-fast centrifuge tube, centrifuged at 100,000 g for 60 min, washed and precipitated with PBS, and centrifuged again under the same conditions. Finally, the precipitate was re-suspended with an appropriate amount of PBS and stored at -80°C.

The morphology of BMSCs-Exo was observed by transmission electron microscopy (TEM, H7650, Hitachi, Japan). The 20µL exosome suspension was absorbed by pipette and drop it on the copper net loaded with carbon membrane for 5-10 min natural adsorption. Then, the excess liquid is sucked away by filter paper, making it semi-dry. Absorb 20µL 2% phosphotungstic acid solution with a pipette, and add it to the copper net for negative staining for 3 min. Excess dye solution was removed with filter paper and dry it under infrared lamp. The copper mesh was observed and photographed under TEM.

The particle size distribution of BMSCs-Exo was detected by dynamic light scattering method using Zetasizer Nano laser nanometer particle sizer (Malvern Zetasizer Nano ZS90, U.K.).

Western blot was conducted to identify the specific protein expression of BMSCs-Exo. After fully cracking BMSCs-Exo, the protein concentration was quantified based on the BCA kit instructions. After electrophoresis, the protein samples were transferred to the PVDF membrane and hatched with 5% skim milk at 25°C for 4 h. HSC70 (1:500), TSG101 (1:500), CD9 (1:1000), and CRP94 (1:500) were incubated at 4°C overnight, and then incubated with the corresponding WB secondary antibody (1×TBST dilution) at room temperature for 2 h. Finally, ECL gel imaging system was used for color development.

### Cell treatment and grouping

In order to study the effect of BMSCs-Exo on osteoblasts, MC3T3-E1 cells were cultured in the complete culture medium containing BMSCs-Exo. Cells were divided into control group (MC3T3-E1 cells were cultured routinely), PBS group (PBS was used instead of culture medium), and BMSCs-Exo group (the culture medium contained 5 µg/mL BMSCs-Exo). To investigate the effect of KLF3-AS1 on MC3T3-E1 cells, MC3T3-E1 cells were transfected with si-KLF3-AS1, and then cultured in a medium containing BMSCs-Exo.

MC3T3-E1 (3 × 10^5^ cells/mL) were inoculated into 6-well plates. According to the protocol and procedure provided in the product description, si-negative control (si-NC) or si-KLF3-AS1 was transfected into cells using Lipofectamine 3000 (Invitrogen, USA).

### Quantitative real‑time PCR

The relative expression levels of KLF3-AS1 and miR-338-3p were detected by qRT-PCR according to the published literatures [15]. Total RNA was extracted from exosomes or cells using TRIzol reagents. The concentration of total RNA was determined by the spectrophotometer NanoDrop2000. The RNA used in the experiment ensured that the ratio of A260/A280 was in the range of 1.8–2.1. The RNA was reverse-transcribed into cDNA according to the instructions of the TaKaRa reverse transcription kit. Subsequently, the cDNA was amplified using qRT-PCR according to the following ratio. Reaction system: 10µL SYBR Premix Ex Taq, 0.6µL cDNA, 7.8µL H_2_O, 0.4µL forward primer, 0.4µL reverse primer. Reaction conditions: pre-denaturation at 90°C for 30s (1 cycle), with 40 cycles of denaturation at 95°C for 5s, annealing and extension at 60°C for 20s. According to the obtained Ct value, the expression difference multiples between the experimental group and the control group were calculated. Relative expression was calculated by 2^−△△Ct^ method. Primers required for this experiment are as follows: KLF3-AS1: forward primer: 5’-CTGTAGGCGCGCTCTTTC-3’, KLF3-AS1: reverse primer: 5’-TCCGACCAAAGTTTGCCAAG-3’; GAPDH: forward primer: 5’-CTGCACCACCAACTGCTTAG-3’; reverse primer: 5’-AGGTCCACCACTGACACGTT-3’; miR-338-3p: forward primer: 5’-GCGTCCAGCATCAGTGATT-3’, reverse primer: 5’-GTGCAGGGTCCGAGGT-3’; U6: forward primer: 5’-GCTCGCTTCGGCAGCACA-3’, reverse primer: 5’-GAGGTATTCGCACCAGAGGA-3’.

### Cell viability

The cell viability tests were evaluated by MTT method, and the experimental procedures were carried out according to the previously published methods [16]. In brief, MC3T3-E1 cells were inoculated in 96-well plate at the density of 1 × 10^5^ cell/well. According to the experimental procedure, the corresponding BMSCs-Exo was put into the cells and cultured for 24 h. Subsequently, 20µL MTT solution was added into each well and incubated for 4 h, dimethyl sulfoxide (DMSO) was added to each well. Microplate reader (Thermo Fisher Scientific, USA) measured OD value at 490 nm and cell viability was calculated.

### Cell migration

Cell migration was assessed by Transwell assay based on the published methods [17]. The experimental procedure follows the previously published literature with minor modifications. In short, MC3T3-E1 cells were added into the Transwell chamber (2 × 10^5^ cells/mL), and complete culture medium, PBS and BMSCs-Exo were respectively added to chamber based on the experimental procedure. Culture medium containing exosome-free FBS was added to the lower layer of Transwell. After incubation at 37°C for 48 h, the chamber was taken out, washed twice with PBS, fixed with 4% paraformaldehyde solution for 10 min, and then stained with 0.1% crystal violet for 1 min. Finally, the cell migration was observed with fluorescence microscope, and 5 fields were randomly selected to take photos and count.

### Cell apoptosis

Cell apoptosis was evaluated by flow cytometry and Annexin V-FITC/PI double staining based on previous published study [18]. MC3T3-E1 cells (5 × 10^3^ cells/well) were inoculated into 24-well plates. After being treated with the corresponding BMSCs-Exo, 5µL FITC and 5µL PI were added in turn according to the instructions of Apoptosis detection kit and incubated at room temperature for 1 h away from light. Finally, the staining agent was discarded, and cold PBS was added to the cells. The cell apoptosis rate was determined using flow cytometer (BD biosciences, USA).

### Dual‑luciferase reporter assay

The online database StarBase v2.0 was used to predict the targeting relationship between miR-338-3p and KLF3-AS1, and the luciferase reporter gene was used to further verify the predicted results. The 3’-UTR sequence of KLF3-AS1 was obtained from the gene bank, and GenePharma (Shanghai, China) was commissioned to synthesize the 3’-UTR wild-type nucleotide sequence of KLF3-AS1 including the binding site of miR-338-3p, while the mutant sequence of the 3’-UTR wild-type sequence of KLF3-AS1 was mutated at the binding site of miR-338-3p. The amplified product was inserted into the SacI and Xhol restriction sites of a pmirGLO vector to produce a WT and MUT pmirGLO vector. WT-KLF3-AS1 and MUT-KLF3-AS1 were co-transfected into MC3T3-E1 cells with miR-338-p mimics or inhibitors using Lipofectamine 3000. 48 h later, the cells were harvested, and the luciferase activity was measured with the Dual-Luciferase Reporter Assay System (Promega). Relative luciferase activity = firefly luciferase activity/renilla luciferase activity.

### Gene Ontology (GO) and Kyoto Enrichment of Genes and Genomes (KEGG) pathway enrichment analysis

The GO and KEGG pathway enrichment of miR-338-3p regulated target genes was analyzed using the DAVID database. GO analysis annotates target genes of miR-338-3p based on three aspects: biological process (BP), cell component (CC), and molecular function (MF). KEGG pathway enrichment analysis is used to study gene function and pathways in large datasets from high-throughput experiments. *P* < 0.05 was considered a significant difference.

### Construction and analysis of protein-protein interaction (PPI) networks

To further study the potential molecular mechanism of miR-338-3p, PPI network analysis was conducted. All target genes of miR-338-3p were imported into the STRING database to obtain the interaction relationship among all target genes.

### Data analysis

SPSS 21.0 and GraphPad Prism 7.0 were used for data analysis and image drawing. Quantitative data conforming to normal distribution were expressed as mean ± standard deviation (SD). Independent T-test was used to compare the two groups of data, and one-way ANOVA and Tukey post-hoc test were used to compare the data between multiple groups. Data at different time points were compared between groups using repeated analysis of variance. *P* < 0.05 was considered as significant difference.

## Results

### Serum KLF3-AS1 expression in patients with fracture is up-regulated

**Fig. 1:**
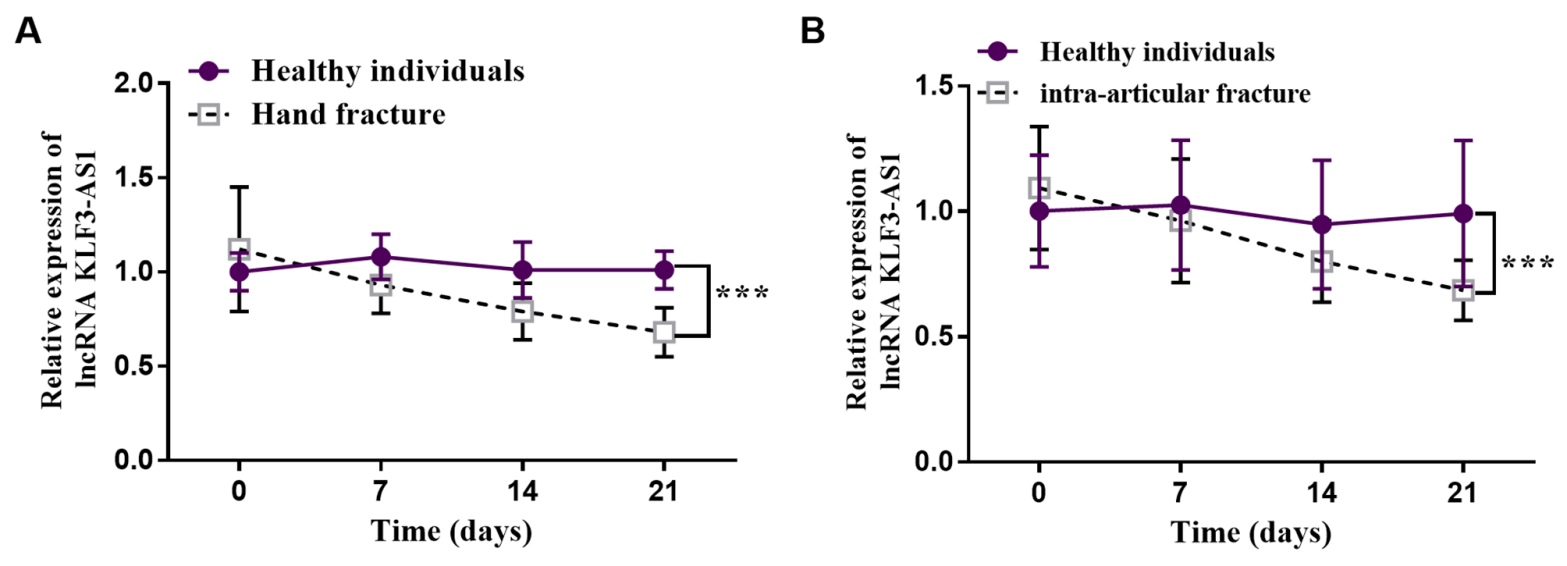
The expression level of serum KLF3-AS1. Serum KLF3-AS1 expression was down-regulated in patients with **(A)** hand and **(B)** intraarticular fractures (Healthy individuals = 65, Hand fracture = 35, Intra-articular fracture = 30). ^***^p < 0.001 vs. Healthy individuals (Independent sample t test)

The expression level of KLF3-AS1 in subjects’ serum was detected using qRT-PCR. Figure 1A showed that the expression level of KLF3-AS1 gradually decreases with an increase in healing time in patients with hand fractures (*p* < 0.001). The same phenomenon can be observed in patients with intra-articular fracture. Figure 1B demonstrated a significant decrease in the expression level of KLF3-AS1 in patients with intra-articular fracture compared to the control group at day 21 post-fracture (*p* < 0.001). Based on these findings, it is hypothesized that dysregulation of KLF3-AS1 may exert a regulatory role in the process of fracture healing.

### Characterization of exosomes

**Fig. 2:**
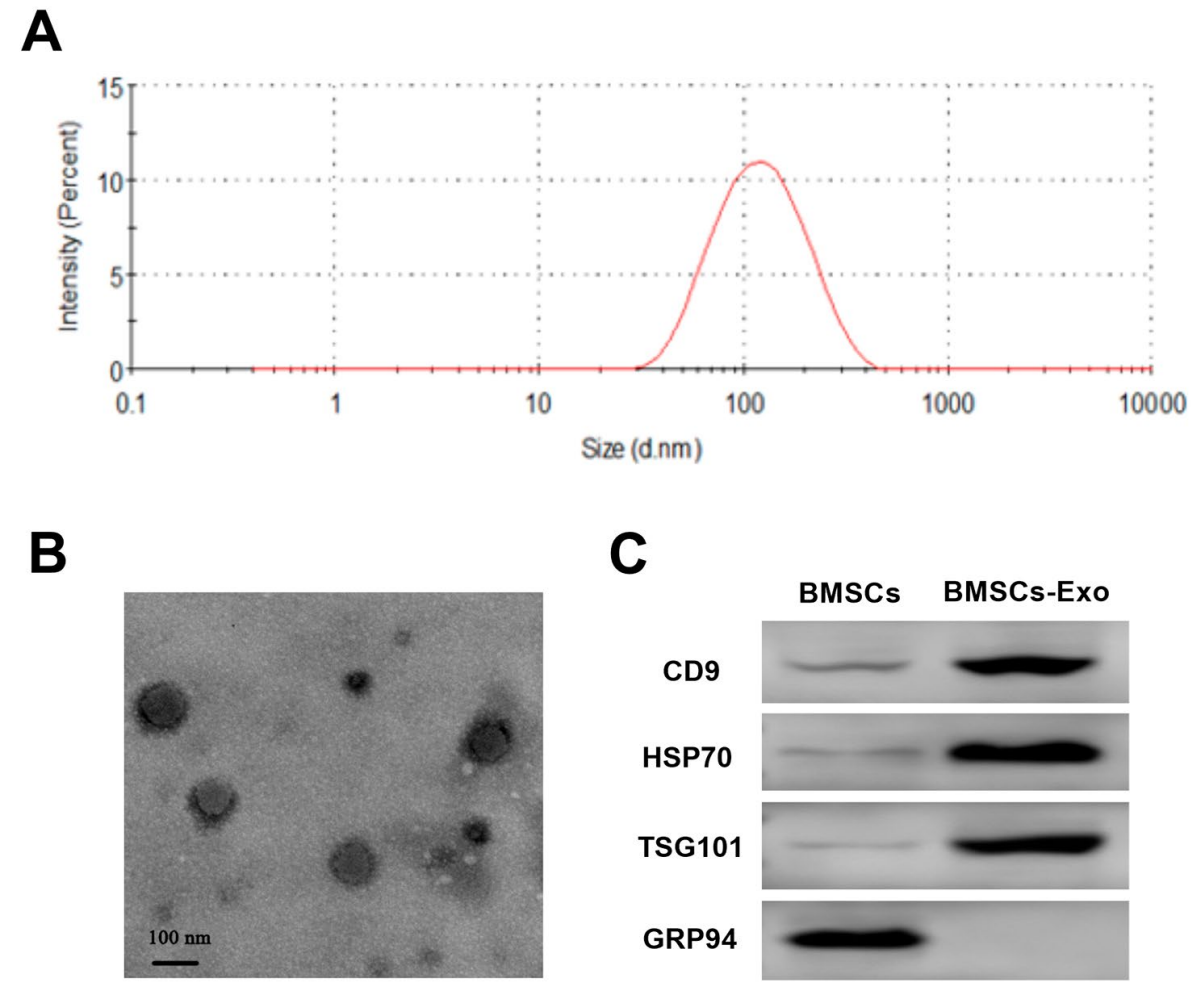
Identification of BMSCs-exosomes. **(A)** Size distribution of BMSCs-exosomes. **(B)** transmission electron microscopy image of BMSCs-exosomes. Scale bars: 100 nm. **(C)** Western blot results of exosome surface proteins

The morphology and particle size of BMSCs-Exo were analyzed, as shown in Fig. 2. The Malvern laser nanosizer analyzer showed an average particle size of approximately 100 nm for BMSCs-Exo (Fig. 2A). TEM analysis demonstrated a uniform spherical structure for BMSCs-Exo (Fig. 2B). Western blot results presented in Fig. 2C indicated increased expression of exosome-specific marker protein TSG101, CD9 and HSP70 in BMSCs-Exo lysates compared to BMSCs lysates. Furthermore, the endoplasmic reticulum marker GRP94 was predominantly expressed in BMSCs but minimally detected in BMSCs-Exo.

### BMSCs-Exo promoted the proliferation and migration of MC3T3-E1 cells and inhibited their apoptosis

**Fig. 3:**
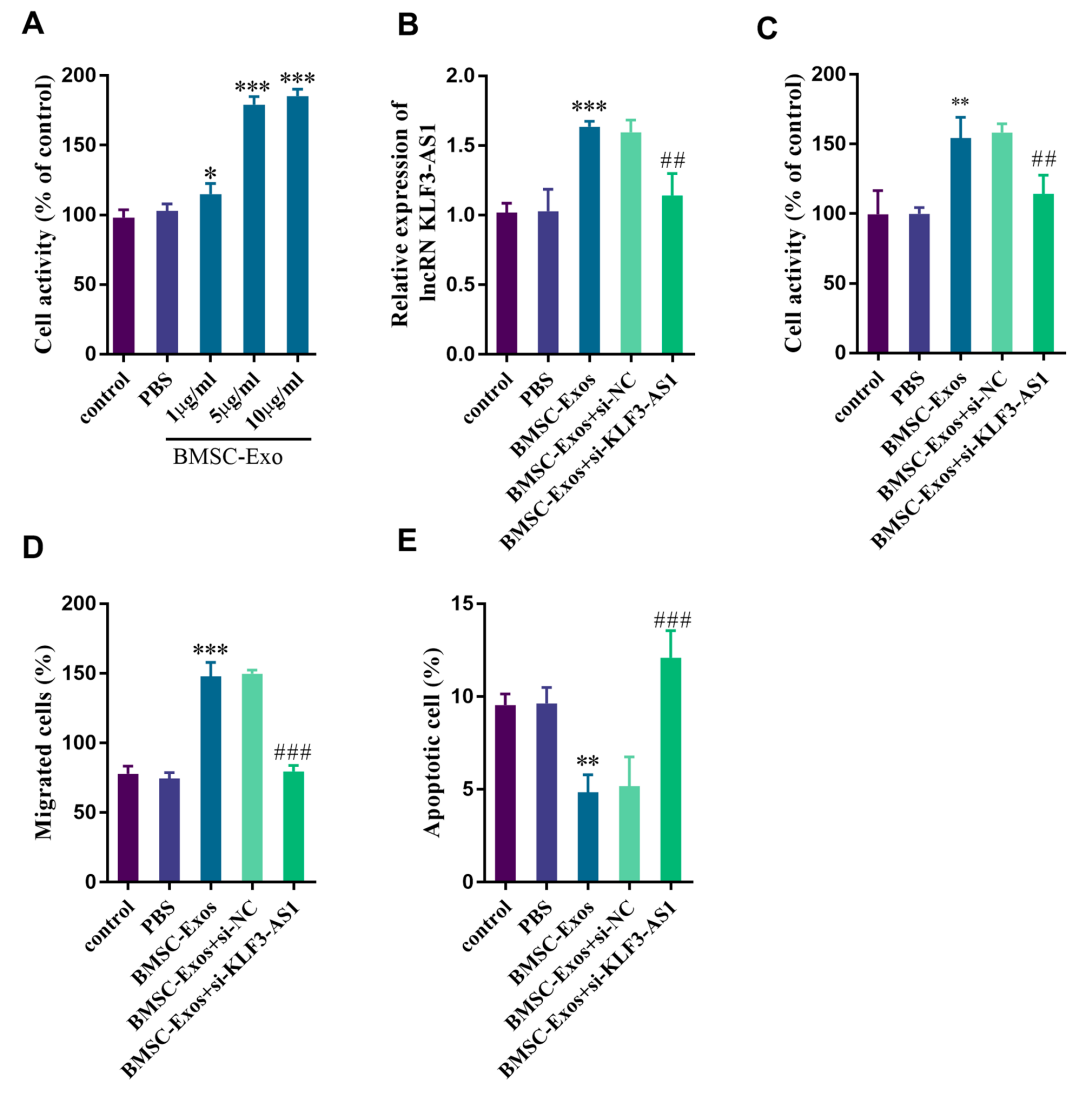
Effects of BMSCs-exosomes on the function of MC3T3-E1 cells. **(A)** Screening of BMSCs-Exo concentration. **(B)** BMSCS-EXO-induced KLF3-AS1 elevation can be transfected with si-KLF3-AS1 inversion. BMSCs-Exo can promote the **(C)** cell viability and **(D)** migration of MC3T3-E1 cells and inhibit **(E)** cell apoptosis. ^*^p < 0.05, ^**^p < 0.01, ^***^p < 0.001 vs. control group; ^##^*p* < 0.01, ^###^*p* < 0.001 vs. BMSCs-Exo group (*n* = 5, one-way ANOVA).

In order to determine the optimal concentration of BMSCs-Exo for cell screening, the effects of different concentrations of BMSCs-Exo on the viability of MC3T3-E1 cells were evaluated. The results revealed no significant difference in cell viability between conventional culture and PBS culture. However, it is noteworthy that MC3T3-E1 cell viability gradually enhanced with the increase of BMSCs-Exo concentration. Since there was no significant difference in promoting cell viability between 5 µg/mL and 10 µg/mL BMSCs-Exo, 5 µg/mL BMSCs-Exo was selected as the experimental concentration (Fig. 3A, *p* < 0.001). Compared to conventional culture and PBS culture, the expression level of KLF3-AS1 significantly increased in MC3T3-E1 cells cultured with BMSCs-Exo. However, transfection of si-KLF3-AS1 into MC3T3-E1 cells did not result in a significant upregulation of KLF3-AS1 expression in BMSCs-Exo cultured cells (Fig. 3B, *p* < 0.01). Regarding cellular function regualtion, BMSCs-Exo culture promoted MC3T3-E1 cell viability and migration while reducing cell apoptosis. Nevertheless, after transfection with si-KLF3-AS1 inhibiting KLF3-AS1 expression in MC3T3-E1 cells, there was no significant regulatory effect on cellular function observed during BMSCs-Exo culturing (Fig. 3C-E, all *p* < 0.05). This outcome may be attributed to the ability of BMSCs-Exo to regulate KLF3-AS1 expression thereby enhancing cell viability and migration while inhibiting apoptosis.

### MiR-338-3p is a target gene of KLF3-AS1

**Fig. 4:**
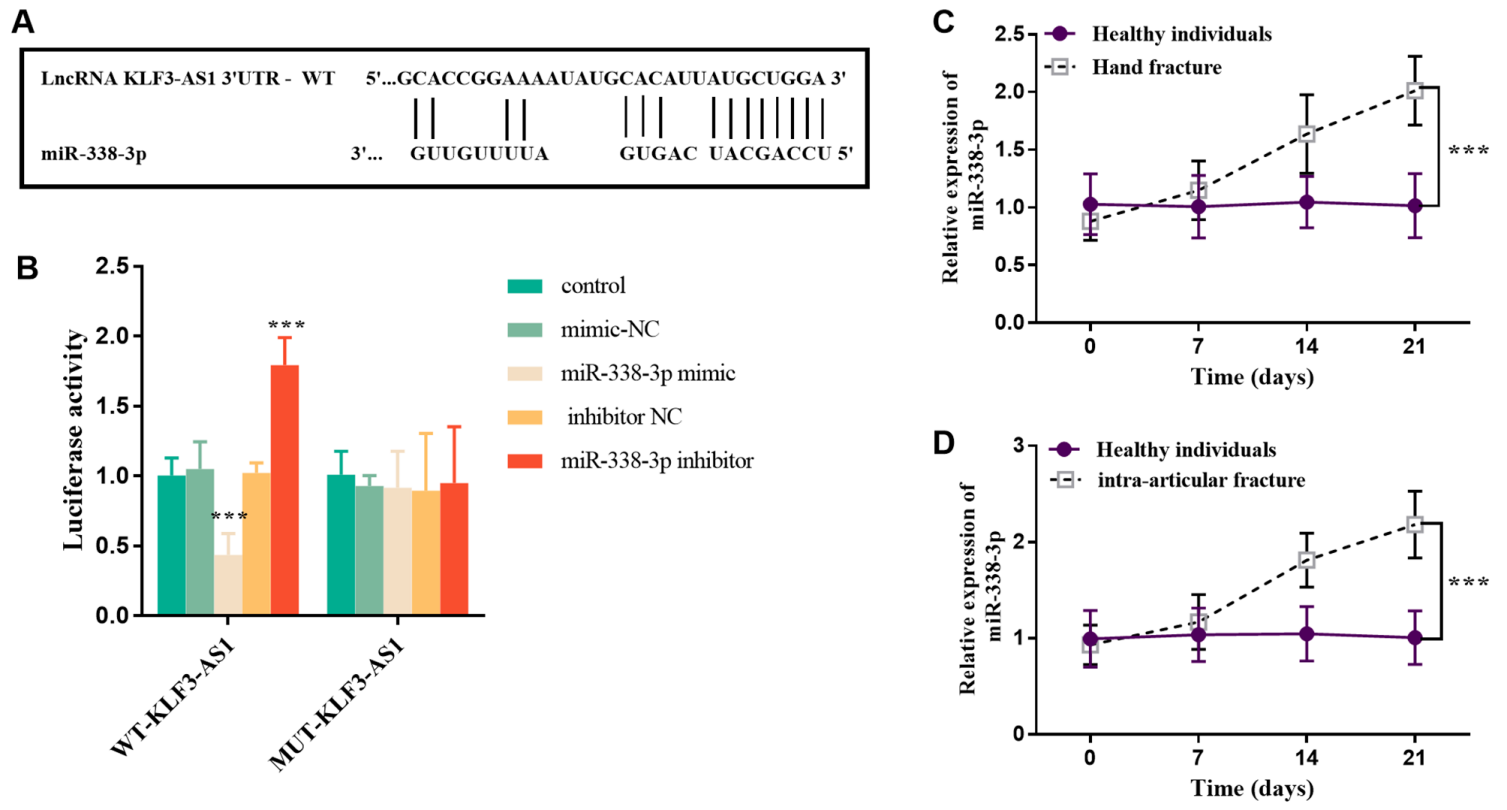
miR-338-3p has a targeting relationship with KLF3-AS1. **(A)** Complementary sites of miR-338-3p and KLF3-AS1. **(B)** luciferase reporter gene. ^***^p < 0.001 vs. Control group (*n* = 5, one-way ANOVA). The expression level of miR-338-3p in serum was up-regulated in patients with **(C)** hand fracture and **(D)** intraarticular fracture (Healthy individuals = 65, Hand fracture = 35, Intra-articular fracture = 30). ^***^p < 0.001 vs. Healthy individuals (Independent sample t test)

StarBase v2.0 predicted complementary binding sites between miR-338-3p and KLF3-AS1 with their respective sequences shown in Fig. 4A. The luciferase reporter gene verified the targeting relationship between miR-338-3p and KLF3-AS1. Transfection with mimic or inhibitor of miR-338-3p resulted in decreased or increased luciferase activity respectively in the WT group but had no effect on luciferase activity in the MUT group indicating specific binding between miR-338-3p and KLF3-AS1 (Fig. 4B, *p* < 0.001). Furthermore, analysis of serum samples from patients revealed a significant upregulated trend of miR-338-3p during fracture healing compared to healthy individuals both with hand fractures and intra-articular fractures (Fig. 4C-D, *p* < 0.001).

### Down-regulation of miR-338-3p can inhibit the promotion of BMSCs-Exo on the proliferation and migration of MC3T3-E1 cells and the inhibition of cell apoptosis

**Fig. 5:**
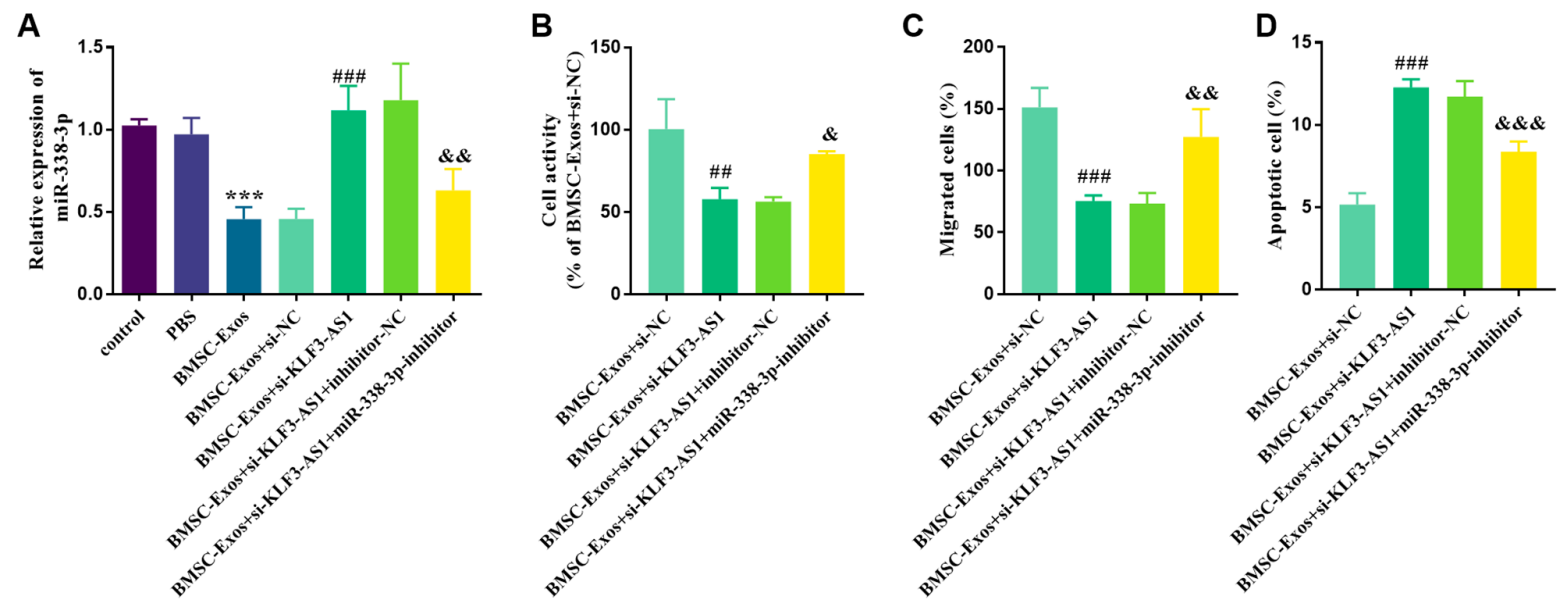
Effects of miR-338-3p in MC3T3-E1 cells incubated with BMSCs-Exo. **(A)** Transfection of miR-338-3p inhibitors down-regulated the expression level of miR-338-3p in BMSCs-Exo incubated MC3T3-E1 cells. The decrease of miR-338-3p can promote the **(B)** viability and **(C)** migration of BMSCs-Exo incubated MC3T3-E1 cells, and inhibit **(D)** cell apoptosis. ^***^p < 0.001 vs. Control group; ^##^*p* < 0.01, ^###^*p* < 0.001 vs. BMSC-Exos + si-NC group; ^&^*p* < 0.05, ^&&^*p* < 0.01, ^&&&^*p* < 0.001 vs. BMSC-Exos + si-KLF3-AS1 + inhibitor-NC group (*n* = 5, one-way ANOVA).

As shown in Fig. 5A, the expression level of miR-338-3p in MC3T3-E1 cells cultured with BMSCs-Exo was down-regulated compared with those cultured with conventional culture or PBS (*p* < 0.001). After si-KLF3-AS1 was transfected into MC3T3-E1 cells cultured in BMSCs-Exo, the expression level of miR-338-3p increased (*p* < 0.001). At the same time, the expression level of intracellular miR-338-3p was declined after BMSCs-Exo incubation after transfection of si-KLF3-AS1 and miR-338-3p inhibitor into MC3T3-E1 cells (*p* < 0.01). Furthermore, as shown in Fig. 5B-D, intracellular transfection of si-KLF3-AS1 down-regulated the expression of KLF3-AS1, inhibited the viability and migration of MC3T3-E1 cells and enhanced apoptosis. However, after transfection of miR-338-3p inhibitor, cell viability and migration ability were up-regulated, while apoptosis was down-regulated (*p* < 0.05).

### Function analysis of mir-338-3p candidate genes

**Fig. 6:**
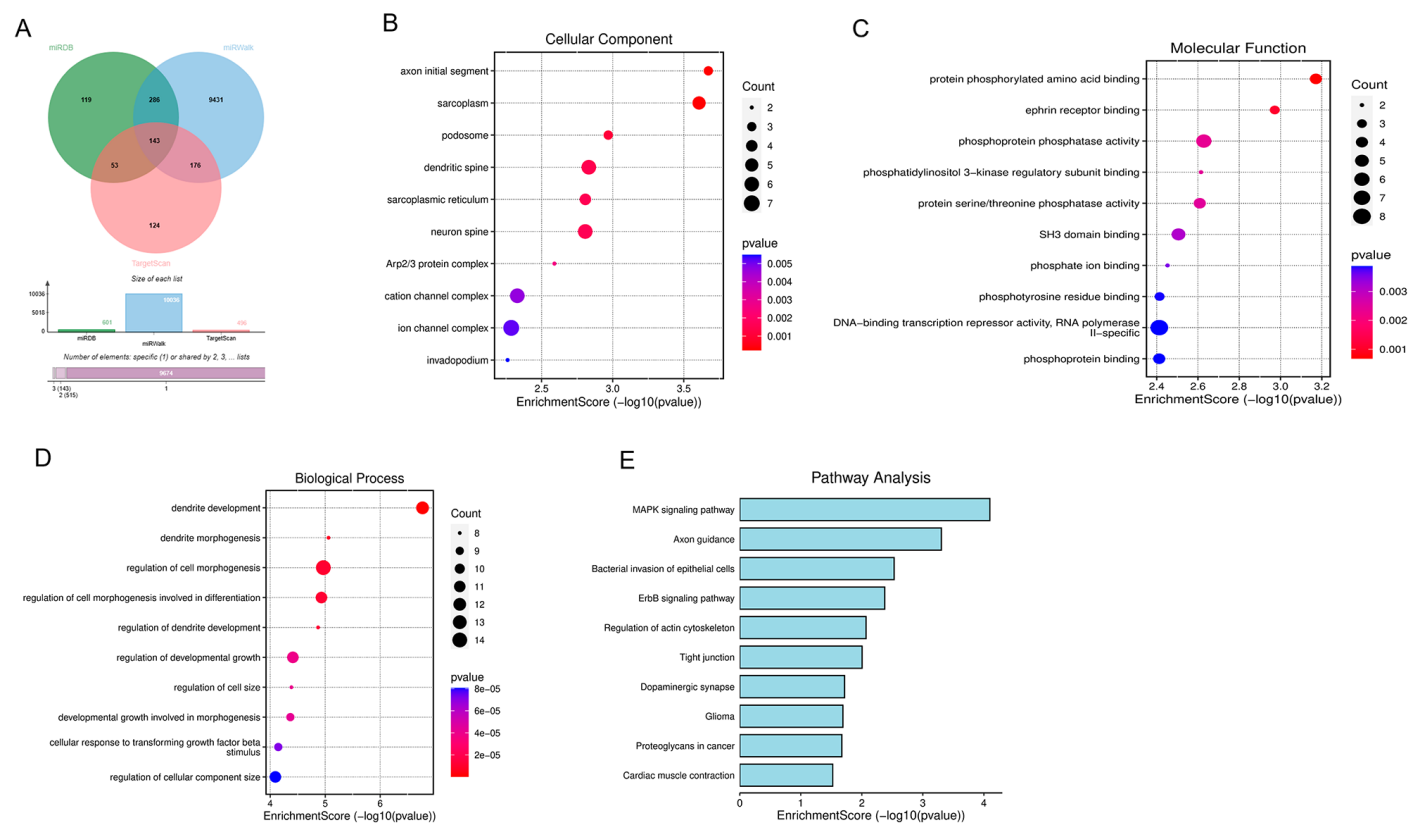
Bioinformatics analysis. **(A)** Venn diagram of miRDB, miRWalk and TargetScan to predict the target genes of miR-338-3p. GO enrichment analysis of target genes of miR-338-3p, **(B)** Cellular component, **(C)** Molecular function and **(D)** Biological process. **(E)** KEGG pathway analysis for target genes of miR-338-3p

**Fig. 7:**
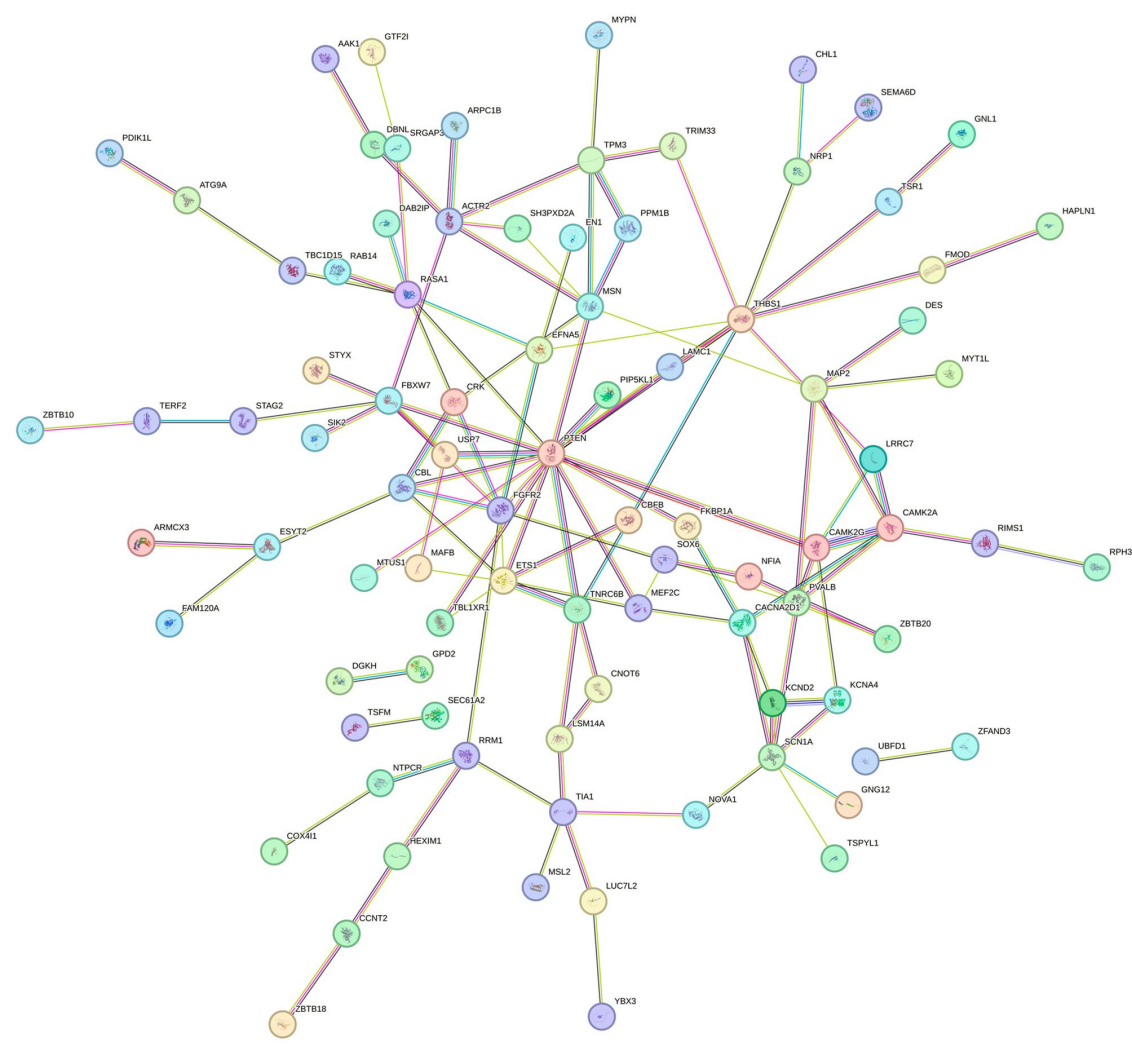
Protein-protein interaction networks for target genes of miR-338-3p

Figure 6A illustrates the predicted target genes of miR-338-3p in miRDB, TargetScan, and miRWalk databases as 601, 496, and 10,036 respectively. A total of 143 target genes were predicted by at least three databases. GO enrichment analysis was performed on the target genes of miR-338-3p. The target genes of miR-338-3p mainly localize to the initial segment of axons and are involved in molecular functions such as protein phosphorylation, amino acid binding, adrenergic receptor binding, phosphatase activity, and protein serine/threonine phosphatase activity (*p* < 0.05), as depicted in Fig. 6B-D. In addition, these target genes of miR-338-3p are involved in a variety of biological processes including regulation of development and growth, dendritic morphogenesis, and cell size regulation (*p* < 0.05). KEGG pathway analysis revealed enrichment of MAPK signaling pathway and ErbB signaling pathway among the target genes (Fig. 6E, *p* < 0.05). Additionally, a total of 143 target genes were submitted to STRING database, the PPI networks was shown in Fig. 7. Table 1 summarized the top 10 high-score gene names derived from this network analysis.

**Table 1:** The top 10 nodes of delayed fracture union-related PPI network

Node	Description	Degree
PTEN	Phosphatase and tensin homolog	16
THBS1	Thrombospondin-1	9
ETS1	Protein C-ets-1	8
FGFR2	Fibroblast growth factor receptor 2	8
FBXW7	F-box/WD repeat-containing protein 7	7
MSN	Moesin	7
RASA1	Ras GTPase-activating protein 1	7
SCN1A	Sodium channel protein type 1 subunit alpha	7
ACTR2	Actin-related protein 2	6
CAMK2A	Calcium/calmodulin-dependent protein kinase type II subunit alpha	6

### PTEN is the target gene of mir-338-3p in MC3T3-E1 cells

**Fig. 8:**
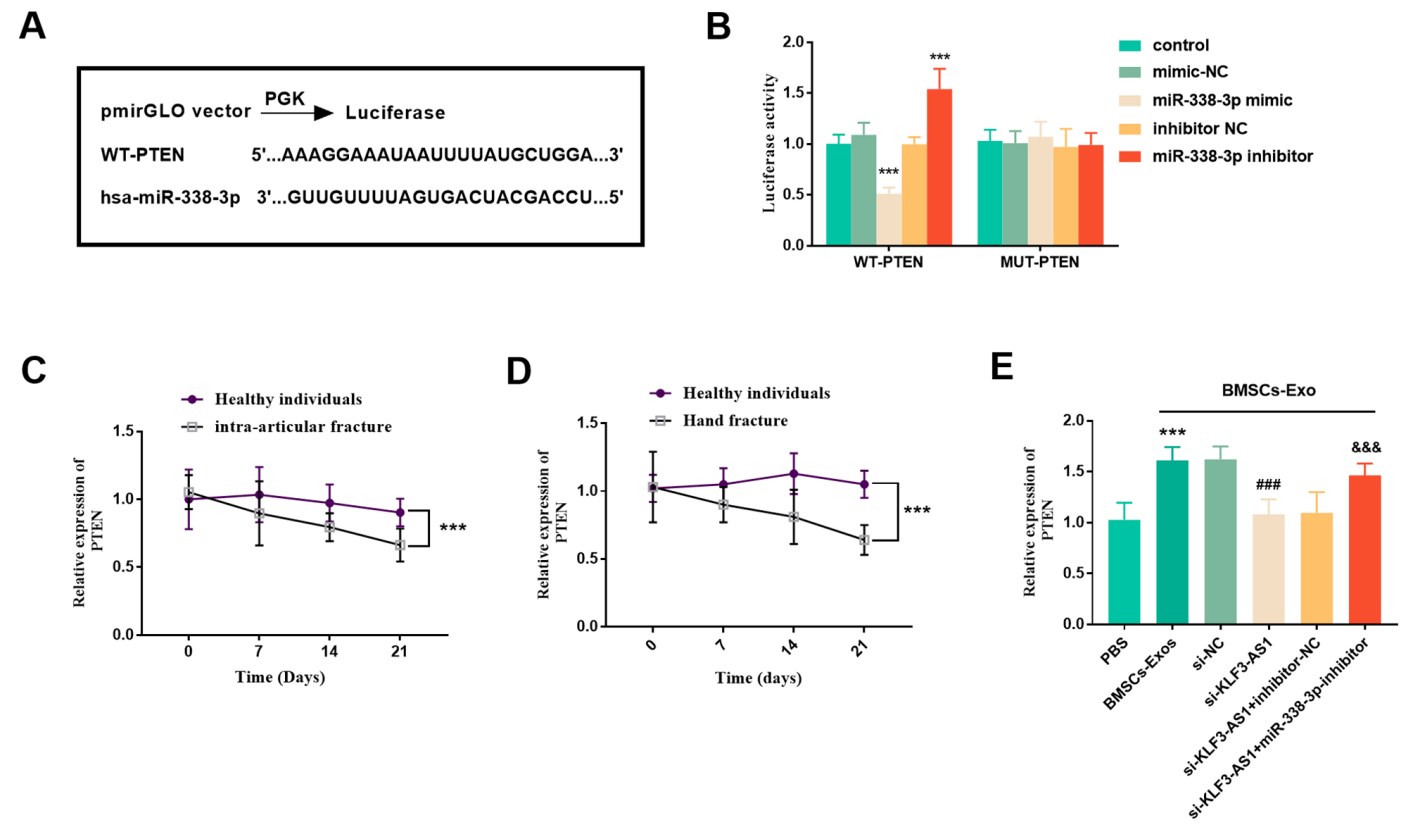
PTEN is a target gene of miR-338-3p. **(A)** Complementary sites of miR-338-3p and PTEN. **(B)** luciferase reporter gene assay. ^***^p < 0.001 vs. Control group (*n* = 5, one-way ANOVA). The expression level of PTEN in serum was down-regulated in patients with **(C)** hand fracture and **(D)** intraarticular fracture (Healthy individuals = 65, Hand fracture = 35, Intra-articular fracture = 30). ^***^p < 0.001 vs. Healthy individuals (Independent sample t test). **(E)** The content of PTEN was increased in BMSCs-Exo incubated cells. ^***^p < 0.001 vs. control group; ^###^*p* < 0.001 vs. BMSCs-Exo + si-NC group; ^&&&^*p* < 0.001 vs. BMSCs-Exo + si-KLF3-AS1 + inhibitor-NC group (*n* = 5, one-way ANOVA).

Figure 8A showed the complementary binding sites of PTEN and miR-338-3p. Subsequently, luciferase experiments were performed in MC3T3-E1 cells. The results showed that miR-338-3p mimics or inhibitors could decrease and enhance luciferase activity in WT group, respectively, but had no effect on MUT group (Fig. 8B, *P* < 0.001). Next, reduced expression of PTEN was found in serum from patients with both hand fracture and intra-articular fracture in clinical samples (Fig. 8C-D, *P* < 0.001). Furthermore, in cell experiments, increased PTEN expression was observed in BMSCs-Exo cultured cells. After pre-inhibition of intracellular KLF3-AS1, the expression of intracellular PTEN decreased, while after simultaneous inhibition of KLF3-AS1 and miR-338-3p, the expression of PTEN increased (Fig. 8E, *P* < 0.001).

## Discussion

In this study, the expression levels of serum KLF3-AS1 and miR-338-3p in patients with fracture exhibited contrasting trends, with an increase and decrease, respectively, indicating dysregulation of both KLF3-AS1 and miR-338-3p following fracture or prior to fracture healing. In vitro experiments demonstrated that BMSCs-Exo promoted the proliferation and migration of osteoblasts while inhibiting apoptosis. Subsequent investigations revealed upregulation KLF3-AS1 expression in MC3T3-E1 cells incubated with BMSCs-Exo compared to the conventional control group, whereas miR-338-3p expression was down-regulated. Further inhibition of KLF3-AS1 in MC3T3-E1 cells abolished the effects of BMSCs-Exo on promoting osteoblasts proliferation and migration as well as inhibiting apoptosis. The luciferase reporter gene assay confirmed the targeting relationship between KLF3-AS1 and miR-338-3p in MC3T3-E1 cells.

Exosomes have garnered significant attention in the field of bone tissue repair and regeneration over the past decade, as they hold potential for restoring or enhancing bone injury, improving dysfunction, and repairing missing tissue [19]. Qin et al. conducted in vivo functional analysis on rats with skull defects and observed that BMSCs-Exo significantly augmented osteoblast regenerative ability [20]. Furuta et al. reported that injection of BMSCs-Exo expedited hypertrophic chondrocyte formulation, woven bone deposition, and vascularization, thereby reversing delayed bone healing [21]. Chia et al. implanted exosomes into the backs of nude mice and demonstrated enhanced vascularization and calcium phosphate formation by stents containing exosomes compared to control stents [22]. Our study revealed that incubation with BMSCs-Exo promoted the proliferation and migration of osteoblasts while inhibiting apoptosis. Furthermore, we investigated the underlying mechanism through which BMSCs-Exo facilitates osteoblast proliferation. We found a significant upregulation of KLF3-AS1 expression in MC3T3-E1 cells incubated with BMSCs-Exo. Fracture healing necessitates a substantial number of new osteoblasts to accelerate bone formation, calcification, increase bone volume and density. LncRNAs are involved in various biological processes such as tumorigenesis, embryonic growth, and stem cell differentiation. Some lncRNAs have been shown to regulate fracture healing. For instance, Guo et al. reported that SNHG1 inhibits osteoblast proliferation by down-regulating the level of miR-181-5p leading to impaired tibial fracture healing [23]. Gu et al. found reduced KCNQ1OT1 expression in patients with delayed fracture healing, and it could induce osteoblast proliferation and inhibit apoptosis through activation of the Wnt/β-catenin signaling pathway, thus accelerating fracture healing [24]. Dysregulated expression of KLF3-AS1 has been found in many diseases, including decreased expression in esophageal cancer and increased expression in myocardial infarction [25, 26]. In our study, KLF3-AS1 was found to be down-regulated in patients with fractures, specifically hand fractures and intra-articular fractures. In addition, we observed increased expression of KLF3-AS1 in MC3T3-E1 cells following BMSCs-Exo treatment during in vitro studies. However, silencing KLF3-AS1 inhibited the rescue ability of BMSCs-Exo on MC3T3-E1 cells. Therefore, we hypothesized that BMSCs-Exo mediated the effects of KLF3-AS1 to promote osteoblast proliferation, migration, and apoptosis inhibition.

The most extensively studied mechanism for lncRNA is their role as miRNA sponge which competitively adsorb miRNAs to block their inhibitory effect on mRNA expression and thereby positively regulate mRNA levels [27, 28]. For example, H19 acts as a competitive sponge for miR-141 during the osteogenic differentiation of rat ectodermal mesenchymal stem cells to promote the expression of osteogenic markers ALP and OCN by blocking β-catenin inhibition [8]. In this study, we demonstrated that KLF3-AS1 functions as a molecular sponge for miR-338-3p and its down-regulated was observed in BMSCs-Exo incubated MC3T3-E1 cells. MiR-338-3p is significantly associated with osteogenic diseases, it was significantly elevated during osteoclast differentiation [29] and elevated in aged rat bone marrow-derived mesenchymal stem cells where it may regulate PCSK5 to inhibit age-related osteoporosis [12]. Consistently with these findings, we also observed an increase in serum levels of miR-338-3p among patients with fracture. Moreover, inhibition of KLF3-AS1 resulted significant upregulation of miR‑338‑5p in MC3T3-E1 cells incubated with BMCSs-Exo. Similarly, in this state, the viability and migration capacity of MC3T3-E1 cells were attenuated, while their apoptotic potential was enhanced. These findings suggest that BMSCs-Exo can promote osteoblast proliferation and migration while inhibiting apoptosis through the regulation of KLF3-AS1/miR-338-3p axis.

Here, we further conducted a comprehensive prediction and identification of downstream target genes for miR-338-3p. Our analysis revealed that these target genes of miR-338-3p are significantly enriched in many biological processes including dendrite morphogenesis, metabolism, growth and development regulation, receptor binding, as well as enzyme activity regulation. In KEGG analysis, the target genes of miR-338-3p were mainly located in MAPK and ErbB signaling pathways. Notably, overactivation of MAPK signaling pathway has been strongly associated with neurofibromatosis type 1 development which often presents skeletal weakening symptoms, including osteopenia, osteoporosis, and long bone pseudarthrosis [30]. Additionally, PPI network analysis identified several key genes potentially regulating miR-338-3p expression including PTEN, THBS1, ETS1, FGFR2, FBXW7, MSN, RASA1, SCN1A, ACTR2, CAMK2A. Reviewing relevant literature supports our predictions by demonstrating gradual increase in levels of target genes like as PTEN and THBS1 during fracture healing [31–33].

Limitations of this study are as follows: n the current study, we only evaluated the role of BMSCs-Exo in mouse cell lines, and the role in animals can better illustrate its actual clinical value. In subsequent experiments, mouse fracture models should be constructed to evaluate the effects of BMSCs-Exo and ncRNA on fracture healing. In addition, RNA-seq analysis was not performed in this study, so we cannot know the specific expression of downstream target genes of miR-338-3p predicted in bioinformatics analysis. In future studies, we should try to use RNA-seq to identify ncRNA expression in blood or tissue samples.

In conclusion, BMSCs-Exo mediated by KLF3-AS1 promotes osteoblast proliferation, migration, and inhibit apoptosis via inhibiting miR-338-3p expression. Therefore, BMSCs-Exo-mediated KLF3-AS1 may serve as a promising therapeutic target for bone-related diseases, such as fracture healing.

## Data Availability

The datasets used and/or analysed during the current study are available from the corresponding author on reasonable request.

## References

[1] Zhang L, Jiao G, Ren S, Zhang X, Li C, Wu W, Wang H, Liu H, Zhou H, Chen Y (2020). Exosomes from bone marrow mesenchymal stem cells enhance fracture healing through the promotion of osteogenesis and angiogenesis in a rat model of nonunion. Stem Cell Res Ther.

[2] Clines GA (2010). Prospects for osteoprogenitor stem cells in fracture repair and osteoporosis. Curr Opin Organ Transplant.

[3] Hong P, Yang H, Wu Y, Li K, Tang Z (2019). The functions and clinical application potential of exosomes derived from adipose mesenchymal stem cells: a comprehensive review. Stem Cell Res Ther.

[4] Kawamura Y, Sanchez Calle A, Yamamoto Y, Sato TA, Ochiya T (2019). Extracellular vesicles mediate the horizontal transfer of an active LINE-1 retrotransposon. J Extracell Vesicles.

[5] Xu S, Wu X. miR-134 inhibits chondrogenic differentiation of bone marrow mesenchymal stem cells by targetting SMAD6. Biosci Rep 2019, 39(1). 10.1042/BSR20180921 PMC6356013 30135141

[6] Du L, Yu Y, Ma H, Lu X, Ma L, Jin Y, Zhang H (2014). Hypoxia enhances protective effect of placental-derived mesenchymal stem cells on damaged intestinal epithelial cells by promoting secretion of insulin-like growth factor-1. Int J Mol Sci.

[7] Zhang C, Wang L, Jin C, Zhou J, Peng C, Wang Y, Xu Z, Zhang D, Huang Y, Zhang Y (2021). Long non-coding RNA Lnc-LALC facilitates colorectal cancer liver metastasis via epigenetically silencing LZTS1. Cell Death Dis.

[8] Gong YY, Peng MY, Yin DQ, Yang YF (2018). Long non-coding RNA H19 promotes the osteogenic differentiation of rat ectomesenchymal stem cells via Wnt/beta-catenin signaling pathway. Eur Rev Med Pharmacol Sci.

[9] Liu Y, Lin L, Zou R, Wen C, Wang Z, Lin F (2018). MSC-derived exosomes promote proliferation and inhibit apoptosis of chondrocytes via lncRNA-KLF3-AS1/miR-206/GIT1 axis in osteoarthritis. Cell Cycle.

[10] Zhai B, Xie SC, Zhang J, He JJ, Zhu XQ (2023). Dynamic RNA profiles in the small intestinal epithelia of cats after Toxoplasma Gondii infection. Infect Dis Poverty.

[11] Chen C, Liu L (2022). Silencing of lncRNA KLF3-AS1 represses cell growth in osteosarcoma via miR-338-3p/MEF2C axis. J Clin Lab Anal.

[12] Tong J, Zhang M, Li X, Ren G. MicroRNA–338–3p regulates age–associated osteoporosis via targeting PCSK5. Mol Med Rep 2021, 23(2). 10.3892/mmr.2020.11775 PMC7751475 33313955

[13] Liu H, Sun Q, Wan C, Li L, Zhang L, Chen Z (2014). MicroRNA-338-3p regulates osteogenic differentiation of mouse bone marrow stromal stem cells by targeting Runx2 and Fgfr2. J Cell Physiol.

[14] Liu S, Fan M, Xu JX, Yang LJ, Qi CC, Xia QR, Ge JF (2022). Exosomes derived from bone-marrow mesenchymal stem cells alleviate cognitive decline in AD-like mice by improving BDNF-related neuropathology. J Neuroinflammation.

[15] Fathi E, Vandghanooni S, Montazersaheb S, Farahzadi R (2021). Mesenchymal stem cells promote caspase-3 expression of SH-SY5Y neuroblastoma cells via reducing telomerase activity and telomere length. Iran J Basic Med Sci.

[16] Sun B, Ma Y, Wang F, Hu L, Sun Y (2019). Mir-644-5p carried by bone mesenchymal stem cell-derived exosomes targets regulation of p53 to inhibit ovarian granulosa cell apoptosis. Stem Cell Res Ther.

[17] Zhu J, Liu B, Wang Z, Wang D, Ni H, Zhang L, Wang Y (2019). Exosomes from nicotine-stimulated macrophages accelerate atherosclerosis through miR-21-3p/PTEN-mediated VSMC migration and proliferation. Theranostics.

[18] Qu Q, Liu L, Cui Y, Liu H, Yi J, Bing W, Liu C, Jiang D, Bi Y (2022). Mir-126-3p containing exosomes derived from human umbilical cord mesenchymal stem cells promote angiogenesis and attenuate ovarian granulosa cell apoptosis in a preclinical rat model of premature ovarian failure. Stem Cell Res Ther.

[19] Petho A, Chen Y, George A (2018). Exosomes in Extracellular Matrix Bone Biology. Curr Osteoporos Rep.

[20] Darden DB, Stortz JA, Hollen MK, Cox MC, Apple CG, Hawkins RB, Rincon JC, Lopez MC, Wang Z, Navarro E (2020). Identification of Unique mRNA and miRNA expression patterns in bone marrow hematopoietic stem and progenitor cells after trauma in older adults. Front Immunol.

[21] Chai Y, Tan F, Ye S, Liu F, Fan Q (2019). Identification of core genes and prediction of miRNAs associated with osteoporosis using a bioinformatics approach. Oncol Lett.

[22] Chai YC, Mendes LF, van Gastel N, Carmeliet G, Luyten FP (2018). Fine-tuning pro-angiogenic effects of cobalt for simultaneous enhancement of vascular endothelial growth factor secretion and implant neovascularization. Acta Biomater.

[23] Guo X, Zhang J, Han X, Wang G (2022). LncRNA SNHG1 delayed Fracture Healing via modulating miR-181a-5p/PTEN Axis. J Invest Surg.

[24] Gu H, Li Z, Lv XF, Zhao AB, Zhu MY, Zhang Y (2019). LncRNA KCNQ1OT1 delayed fracture healing through the Wnt/beta-catenin pathway. Eur Rev Med Pharmacol Sci.

[25] Liu JQ, Deng M, Xue NN, Li TX, Guo YX, Gao L, Zhao D, Fan RT (2020). lncRNA KLF3-AS1 suppresses Cell Migration and Invasion in ESCC by Impairing miR-185-5p-Targeted KLF3 inhibition. Mol Ther Nucleic Acids.

[26] Mao Q, Liang XL, Zhang CL, Pang YH, Lu YX (2019). LncRNA KLF3-AS1 in human mesenchymal stem cell-derived exosomes ameliorates pyroptosis of cardiomyocytes and myocardial infarction through miR-138-5p/Sirt1 axis. Stem Cell Res Ther.

[27] Li L, Lai Q, Zhang M, Jia J (2021). Long non-coding RNA DLGAP1-AS1 promotes the progression of gastric cancer via miR-515-5p/MARK4 axis. Braz J Med Biol Res.

[28] Gong R, Li ZQ, Fu K, Ma C, Wang W, Chen JC (2021). Long noncoding RNA PVT1 promotes stemness and Temozolomide Resistance through miR-365/ELF4/SOX2 Axis in Glioma. Exp Neurobiol.

[29] Sun Q, Zhang B, Zhu W, Wei W, Ma J, Tay FR (2019). A potential therapeutic target for regulating osteoporosis via suppression of osteoclast differentiation. J Dent.

[30] Sharma R, Wu X, Rhodes SD, Chen S, He Y, Yuan J, Li J, Yang X, Li X, Jiang L (2013). Hyperactive Ras/MAPK signaling is critical for tibial nonunion fracture in neurofibromin-deficient mice. Hum Mol Genet.

[31] Dittmann KH, Mayer C, Stephan H, Mieth C, Bonin M, Lechmann B, Rodemann HP (2022). Exposure of primary osteoblasts to combined magnetic and electric fields induced spatiotemporal endochondral ossification characteristic gene- and protein expression profiles. J Exp Orthop.

[32] Zheng K, Wang Y (2021). MiR-193a-3p promotes fracture Healing via Targeting PTEN Gene. Mol Biotechnol.

[33] Xiong Y, Cao F, Hu L, Yan C, Chen L, Panayi AC, Sun Y, Zhou W, Zhang P, Wu Q (2019). miRNA-26a-5p accelerates healing via Downregulation of PTEN in Fracture patients with traumatic brain Injury. Mol Ther Nucleic Acids.

